# Supplementary material for: Trait responsiveness to verbal suggestions predicts nocebo responding: A meta‐analysis
Source: Br J Health Psychol. 2024 Dec 20;30(1):e12774. doi: 10.1111/bjhp.12774 (PMC11662164; doi:10.1111/bjhp.12774)
Supplement: Supplementary file 1 — Data S1. [file BJHP-30-0-s001.docx]

**Supplementary methods**

*Search strategy*

PsycInfo, Embase, MEDLINE and PubMed were searched with the following modified strategy (Rooney et al., 2022): nocebo OR "negative placebo" OR "placebo side effect*" OR "psychogenic symptom*" OR "psychogenic illness*". No temporal restrictions were applied.

*Supplementary data extraction*

The following information was extracted from all included articles independently by the same two reviewers (MVS and MH): study information (authors, publication date, journal, title, country); demographic information (sex assigned at birth distributions, age, ethnicity, education); sample (e.g., university students, clinical population, mixed-sample); study design (between-groups, repeated-measures, mixed-methods); symptom (e.g., pain, itch, dyspnoea, dizziness, nausea, motor inhibition, cognitive performance, etc.); methodological details (counterbalancing, participant and experimenter blinding of condition, blinding assessment, randomisation, experimenter sex); sample size (total and for all conditions/groups); expectancy assessed prior to nocebo administration (yes vs. no); negative symptom information type (absence/presence of: textual suggestions, verbal suggestions, non-verbal suggestion, social observation, direct or indirect suggestion type); inert intervention exposure (pill, procedure, nasal spray, cream, injection, other); and nocebo information type (open vs. deceptive)

**Supplementary results**

*Study quality assessment tool*

Items are rated in a binary fashion (0=No; 1=Yes), and computed into a percent score. Items are based on Cochrane criteria and PRISMA recommendations (adapted from (Stein et al., 2023; Thompson et al., 2019; Wieder et al., 2021).

1. Were relevant reliability and validity data of suggestibility and symptom measure presented in the paper?
2. Was there a clear specification of study objectives?
3. Was it clearly described where participants were drawn from (e.g. University etc)?;
4. Was it clearly described how participants were recruited (e.g. advertisement, course credits, volunteers, etc.)?;
5. Was there a clear description of the inclusion and exclusion criteria?;
6. Was a reliable and valid suggestibility scale used?;
7. Was a referenced symptom measure used? (e.g. length of time tolerated pain for; symptom reporting measure);
8. Was the procedure described in enough detail allowing for independent replication?;
9. Was the experimenter blind to suggestibility OR nocebo level prior to assessment of other (nocebo/suggestibility)?;
10. Were precautions implemented to ensure that participants were blind to experimental condition?;
11. Was the experimenter blind to nocebo/control condition?
12. Were relevant participant characteristics adequately described (age, sex, etc.)?;
13. Were the groups comparable in terms of demographics? [Applicable only to between-groups studies.
14. Was the study pre-registered?
15. Are the data freely available on an open data repository? (e.g., Open Science Framework [osg.io]

**Supplementary Table 1.** *Consensus methodological quality ratings per correlation pair (k=13)*

| **Study ID** | **1** | **2** | **3** | **4** | **5** | **6** | **7** | **8** | **9** | **10** | **11** | **12** | **13** | **14** | **15** | **Total** |
| --- | --- | --- | --- | --- | --- | --- | --- | --- | --- | --- | --- | --- | --- | --- | --- | --- |
| Corsi & Colloca, 2017 | 0 | 1 | 0 | 0 | 1 | 1 | 1 | 1 | 1 | 0 | 0 | 1 | NA | 0 | 0 | 50% |
| Di Stefano et al., 2022 | 0 | 1 | 1 | 0 | 1 | 1 | 0 | 1 | 0 | 1 | 1 | 1 | NA | 0 | 0 | 57% |
| Khan et al., 2009 | 0 | 1 | 1 | 1 | 1 | 1 | 1 | 1 | 1 | NA | NA | 1 | NA | 0 | 0 | 75% |
| Leigh et al., 2003 | 0 | 1 | 1 | 1 | 1 | 1 | 1 | 1 | 1 | 1 | 0 | 0 | 1 | 0 | 0 | 66% |
| Sharav et al., 2023 | 0 | 1 | 0 | 1 | 0 | 1 | 1 | 1 | 0 | 0 | 0 | 1 | 0 | 0 | 0 | 40% |
| Winter & Braw, 2021 | 1 | 1 | 1 | 1 | 1 | 1 | 1 | 1 | 1 | 0 | 1 | 1 | 1 | 0 | 0 | 80% |
| Woody et al., 1997a | 0 | 1 | 1 | 1 | 1 | 1 | 1 | 1 | 1 | 1 | NA | 1 | NA | 0 | 0 | 76% |
| Woody et al., 1997b | 0 | 1 | 1 | 1 | 1 | 1 | 1 | 1 | 1 | 1 | NA | 1 | NA | 0 | 0 | 76% |
| Zech et al., 2019a | 0 | 1 | 1 | 1 | 1 | 1 | 1 | 0 | 0 | 0 | 0 | 1 | NA | 0 | 0 | 50% |
| Zech et al., 2019b | 0 | 1 | 1 | 1 | 1 | 1 | 1 | 0 | 0 | 0 | 0 | 1 | NA | 0 | 0 | 50% |
| Zech et al., 2020a | 1 | 1 | 1 | 1 | 1 | 1 | 1 | 1 | 0 | 0 | 0 | 1 | NA | 0 | 0 | 64% |
| Zech et al., 2020b | 1 | 1 | 1 | 1 | 1 | 1 | 1 | 1 | 0 | 0 | 0 | 1 | NA | 0 | 0 | 64% |
| Zech et al., 2022 | 0 | 1 | 0 | 1 | 1 | 1 | 1 | 1 | 0 | 0 | 0 | 1 | NA | 0 | 0 | 50% |

**Supplementary Table 3*.*** *Meta-regression analyses of suggestibility-nocebo correlation in all studies (k=13)*

| **Moderator** | **Δ*Z_r_* [95% CIs]** | ***Z*** | ***p*** | ***I^2^*** |  |
| --- | --- | --- | --- | --- | --- |
| Dyspnoea | -0.08 [-0.60, 0.43] | -0.32 | .74 | 79% | |
| Dizziness | 0.07 [-0.41, 0.56] | 0.31 | .76 | 80% | |
| Nausea | -0.09 [-0.51, 0.32] | -0.46 | .64 | 79% | |
| Motor inhibition | -0.16 [-0.51, 0.17] | -0.95 | .34 | 77% | |
| Cognitive functioning | 0.24 [-0.13, 0.62] | 1.27 | .20 | 76% | |
| Participant blinding | 0.15 [-0.15, 0.45] | 0.98 | .32 | 78% | |
| Suggestibility scale administration context ^a^ | -0.09 [-0.56, 0.47] | -0.37 | .71 | 77% | |
| Baseline as control condition | -0.13 [-0.47, 0.21] | -0.76 | .45 | 77% | |
| Video suggestion | 0.17 [-0.54, 0.19] | -0.94 | .35 | 78% | |
| Non-verbal suggestion | -0.16 [-0.51, 0.17] | -0.95 | .34 | 77% | |
| Direct verbal suggestibility scale | -0.08 [-0.49, 0.33] | -0.38 | .70 | 79% | |
| Hypnotic context | -0.16 [0.54, 0.21] | -0.85 | .39 | 78% | |

*Notes.* For all binary moderators, 0= variable absent, 1= variable present.

*^a^ k=8:* group vs. individually administered suggestibility scale*.*

**Reference list of included papers**

Corsi, N., & Colloca, L. (2017). Placebo and Nocebo Effects: The Advantage of Measuring Expectations and Psychological Factors. *Frontiers in psychology*, *8*, 308. <https://doi.org/10.3389/fpsyg.2017.00308>

Di Stefano, M., Brondino, N., Bonaso, V., Miceli, E., Lapia, F., Grandi, G., Pagani, E., Corazza, G. R., & Di Sabatino, A. (2022). The Perception of Lactose-Related Symptoms of Patients with Lactose Malabsorption. *International journal of environmental research and public health*, *19*(16), 10234. <https://doi.org/10.3390/ijerph191610234>

Khan, A. Y., Baade, L., Ablah, E., McNerney, V., Golewale, M. H., & Liow, K. (2009). Can hypnosis differentiate epileptic from nonepileptic events in the video/EEG monitoring unit? Data from a pilot study. *Epilepsy & behavior: E&B*, *15*(3), 314–317. <https://doi.org/10.1016/j.yebeh.2009.04.004>

Leigh, R., MacQueen, G., Tougas, G., Hargreave, F. E., & Bienenstock, J. (2003). Change in forced expiratory volume in 1 second after sham bronchoconstrictor in suggestible but not suggestion-resistant asthmatic subjects: a pilot study. *Psychosomatic medicine*, *65*(5), 791–795. <https://doi.org/10.1097/01.psy.0000079454.48714.1b>

Sharav, Y., Haviv, Y., & Tal, M. (2023). Placebo or Nocebo Interventions as Affected by Hypnotic Susceptibility. *Applied sciences, 13*(2), 931. <https://www.mdpi.com/2076-3417/13/2/931>

Winter, D., & Braw, Y. (2022). COVID-19: Impact of diagnosis threat and suggestibility on subjective cognitive complaints. *International journal of clinical and health psychology : IJCHP*, *22*(1), 100253. https://doi.org/10.1016/j.ijchp.2021.100253

Woody, E. Z., Drugovic, M., & Oakman, J. M. (1997). A reexamination of the role of nonhypnotic suggestibility in hypnotic responding. *Journal of personality and social psychology, 72*(2), 399–407. <https://doi.org/10.1037/0022-3514.72.2.399>

Zech, N., Scharl, L., Seemann, M., Pfeifer, M., & Hansen, E. (2022). Nocebo Effects of Clinical Communication and Placebo Effects of Positive Suggestions on Respiratory Muscle Strength. *Frontiers in psychology*, *13*, 825839. <https://doi.org/10.3389/fpsyg.2022.825839>

Zech, N., Schrödinger, M., Seemann, M., Zeman, F., Seyfried, T. F., & Hansen, E. (2020). Time-Dependent Negative Effects of Verbal and Non-verbal Suggestions in Surgical Patients-A Study on Arm Muscle Strength. *Frontiers in psychology*, *11*, 1693. <https://doi.org/10.3389/fpsyg.2020.01693>

Zech, N., Seemann, M., Grzesiek, M., Breu, A., Seyfried, T. F., & Hansen, E. (2019). Nocebo Effects on Muscular Performance - An Experimental Study About Clinical Situations. *Frontiers in pharmacology*, *10*, 219. https://doi.org/10.3389/fphar.2019.00219

**References**

Rooney, T., Sharpe, L., Todd, J., Richmond, B., & Colagiuri, B. (2022). The relationship between expectancy, anxiety, and the nocebo effect: a systematic review and meta-analysis with recommendations for future research. *Health Psychol Rev*, 1-28. <https://doi.org/10.1080/17437199.2022.2125894>

Stein, M. V., Heller, M., Chapman, S., Rubin, G. J., & Terhune, D. B. (2023). *Moderators of the nocebo response in controlled experiments: A meta-analysis* <https://www.crd.york.ac.uk/prospero/display_record.php?RecordID=402097>

Thompson, T., Terhune, D. B., Oram, C., Sharangparni, J., Rouf, R., Solmi, M., Veronese, N., & Stubbs, B. (2019). The effectiveness of hypnosis for pain relief: A systematic review and meta-analysis of 85 controlled experimental trials. *Neuroscience and Biobehavioral Reviews*, *99*, 298-310. <https://doi.org/http://dx.doi.org/10.1016/j.neubiorev.2019.02.013>

Wieder, L., Brown, R., Thompson, T., & Terhune, D. B. (2021). Suggestibility in functional neurological disorder: a meta-analysis. *J Neurol Neurosurg Psychiatry*, *92*(2), 150-157. <https://doi.org/10.1136/jnnp-2020-323706>
